# Expressed emotion mediates the association between caregiver relationship closeness and psychological symptoms in people with dementia

**DOI:** 10.1111/psyg.70000

**Published:** 2025-01-21

**Authors:** Xiaoji Liu, Wenping Mo, Reiko Kanaya, Kazue Shigenobu, Keigo Takiue, Eriko Koujiya, Yasushi Takeya, Miyae Yamakawa

**Affiliations:** ^1^ Division of Health Sciences, Graduate School of Medicine Osaka University Osaka Japan; ^2^ Department of Behavioural Neurology and Neuropsychiatry Osaka University United Graduate School of Child Development Osaka Japan; ^3^ Asakayama General Hospital Osaka Japan; ^4^ Department of Nursing, Graduate School of Medicine Gifu University Gifu Japan

**Keywords:** dementia, expressed emotion, mediation, relationship closeness

## Abstract

**Aim:**

This study examines whether expressed emotion (EE) mediates the association between caregiver relationship closeness and behavioural and psychological symptoms of dementia (BPSD).

**Methods:**

We recruited 64 families with dementia in Osaka and collected data for 3 months. Caregiver–patient relationship closeness was assessed using the Relationship Closeness Scale (RCS), and BPSD was measured with the Neuropsychiatric Inventory Questionnaire (NPI‐Q). EE was evaluated using the Family Attitude Scale (FAS).

**Results:**

The results indicated significant correlations between EE, relationship closeness, and BPSD, showing that higher caregiver intimacy levels were associated with lower aggression in dementia people. Mediation analysis revealed that EE significantly and mainly mediated the relationship between RCS and BPSD.

**Conclusions:**

The findings underscore the importance of interventions focused on improving caregiver–patient relationships and managing caregiver emotional expressions to mitigate BPSD. These insights are valuable for developing targeted support programs for dementia care.

## INTRODUCTION

As the population ages globally, the incidence of cognitive dysfunction continues to increase. A recent meta‐analysis showed that the number of people living with dementia doubles every 5 years. In 2015, approximately 15% of people aged 65 years and older had dementia, and this number is expected to reach 7 million by 2025 in Japan.^1^ Most people with dementia rely on family caregivers for home care, placing considerable stress on caregivers and profoundly affecting family relationships.

Among the myriad challenges faced by people with dementia and their caregivers, behavioural and psychological symptoms of dementia (BPSD) represent a pervasive and distressing manifestation. Research indicates that as many as 80% of people with dementia experience BPSD, which not only exacerbates the distress experienced by dementia people and their caregivers but is also linked to an accelerated progression of the disease.^2^, ^3^, ^4^ The burden of managing BPSD falls heavily on family caregivers who encounter challenges stemming from shifts in communication dynamics and relational disruptions, leading to heightened levels of stress and feelings of bereavement.^5^ Moreover, studies have underscored the intricate relationship between the quality of familial relationships and the manifestation of BPSD, highlighting the pivotal role of familial bonds in influencing the behavioural symptoms exhibited by people with dementia.^6^

Currently, there is no definitive cure for BPSD. Pharmacological treatments such as antipsychotics and antidepressants have exhibited efficacy in targeting specific symptoms associated with BPSD.^7^ However, owing to the attendant adverse effects that necessitate meticulous oversight and specialist monitoring,^8^ these therapeutic modalities are typically reserved as the last resource in BPSD management.^9^ Consequently, the mitigation and effective management of BPSD remain pressing concerns in the clinical and caregiving domains.

Expressed emotion (EE), a concept that originated from research on schizophrenia, has emerged as a crucial determinant of caregiver interaction outcomes. EE encompasses the critical comments, hostility, emotional overinvolvement, positive remarks, and warmth expressed by caregivers toward patients.^10^ Furthermore, EE can also predict the BPSD in people with dementia.^11^, ^12^ High levels of EE are likely linked to worse outcomes in patients, including increased behavioural symptoms.^13^ A longitudinal study has demonstrated that EE mediates the adverse effects of neuropsychiatric symptoms on caregiver well‐being, making it a significant factor in dementia care.^14^

Several studies have revealed associations between EE and the quality of the caregiver–patient relationship. Studies have shown that family members are more likely to exhibit high EE in situations when their relationships is poor.^15^, ^16^, ^17^

Factors significantly associated with relationship quality from the family caregiver's perspective was BPSD.^18^ Systematic reviews indicate that BPSD has cumulative effects on caregivers. These impacts contribute to increased stress, difficulties in coping, feelings of isolation, and challenges in interpersonal relationships.^19^ However, strong familial bonds can counteract some of these negative effects. The quality of these relationships can mitigate the impact of daily BPSD on caregiver distress.^20^

While research highlights the importance of family relationships in BPSD, the specific mechanisms remain unclear. This study aims to investigate whether EE mediates the association between close caregiver–patient relationships and BPSD in individuals with dementia. By understanding this association, we aim to provide valuable insights for improving patient care and promoting family health. Additionally, this study offers evidence for designing intervention programs to support healthcare workers in caring for families affected by dementia.

## METHOD

### Study design and participants

Sixty‐four individuals with dementia and their main family caregivers were recruited from dementia group homes, visiting nursing stations, daycare centres, hospital outpatient clinics, and care management offices in Osaka. At the time of recruitment, all people with dementia lived at home. This study was approved by the Institutional Review Board of Osaka University and required written informed consent from all participants. The inclusion criteria were 18 years of age or older and caring for a person with dementia with weekly contact.

The data were collected in two phases. The initial data (T1) were gathered through face‐to‐face or Zoom interviews at the participants' homes, along with a packet of self‐reported psychosocial measures completed on the same day. The second set of data (T2) were collected 2 months after the initial interview, through face‐to‐face or Zoom interviews.

### Measures

#### Family Attitude Scale (FAS)

The FAS is a 30‐item scale that has been translated into Japanese and validated.^21^ Family caregivers report how often each statement is true on a scale ranging from ‘every day’ (score of 4) to ‘never’ (score of 0). The responses are summed to obtain a total score, ranging from 0 to 120.

#### Relationship Closeness Scale (RCS)

This is a six‐item tool, scored on a four‐point Likert scale. It is used to measure the current relationship with care recipients. The total score on the RCS, which ranges from 6 to 24, is obtained by summing the scores of the six individual items.^22^, ^23^ A higher total score indicates stronger or closer relationships within family units.

#### Japanese version of the Neuropsychiatric Inventory Questionnaire (NPI‐Q)

This scale, translated by Hirono *et al*.,^24^ is comparable in reliability and power to the original version and is a useful tool for measuring neuropsychiatric impairment in people with dementia. The NPI‐Q sub‐items include questions that reflect key symptoms. The patients are first asked to answer ‘yes’ or ‘no’ to the questions on these items. If the answer is ‘no’, the patient proceeds to the next sub‐question. The NPI‐Q provides a severity and burden scale for each symptom, and an overall severity and burden scale, which is the sum of the items. The higher the score, the more severe the symptoms and greater the burden of the disease.

### Statistics

We conducted the statistical analyses using SPSS. The skewness, standard error, and kurtosis indicated that all measures were within acceptable ranges for normality. There were no outliers. We also examined the demographic and disease variables related to caregivers and people with dementia outcomes to identify potential confounders. Regression analysis was conducted to predict follow‐up BPSD based on caregiver variables, controlling for previous BPSD and people with dementia baseline measures. Finally, we used PROCESS macro in SPSS to test EE as a partial mediator in the relationship between caregiving and BPSD.^25^ This method of testing mediation uses bootstrapping, a random resampling process that provides confidence intervals (CIs) for the strength of the indirect effects. Evidence of a significant indirect effect is indicated when the CI does not reach zero. In this study, 5000 bootstrap samples were used.

## RESULTS

### Covariates

The relationships between potential demographic covariates and the NPI‐Q measure at both time points were examined, and none were found to be significant.

### Longitudinal measurements

Frequency and percentage of the BPSD reported by people with dementia at T1 and T2 are shown in Table 1. Depression was the most commonly reported condition at both T1 and T2. Only night‐time behaviour was statistically different (*P* = 0.017).

**Table 1:** The Neuropsychiatric Inventory Questionnaire score over 3 months (*N* = 56)

Types of BPSD^a^	T1 frequency (%)	T2 frequency (%)	*P*
Delusion	17 (30.36%)	10 (17.86%)	0.181
Hallucination	14 (25.00%)	10 (17.86%)	0.410
Aggression	26 (46.43%)	22 (39.29%)	0.478
Depression	31 (55.36%)	28 (50.00%)	0.083
Anxiety	17 (30.36%)	19 (33.93%)	0.713
Elation	6 (10.71%)	4 (7.14%)	0.709
Apathy	23 (41.07%)	20 (35.71%)	0.761
Disinhibition	9 (16.07%)	8 (14.29%)	0.871
Irritability	16 (28.57%)	13 (23.21%)	0.905
Aberrant motor activity	14 (25.00%)	9 (16.07%)	0.168
Night‐time behaviour	24 (42.88%)	12 (21.43%)	0.017
Appetite/eating change	28 (50.00%)	23 (41.07%)	0.489
Total	53 (94.64%)	49 (87.50%)	0.068

^a^ Behavioural and psychological symptoms of dementia.

### Correlations

Correlations between the FAS and RCS at T1 and NPI‐Q at T2 are shown in Table 2. There were significant correlations between delusions and the RCS (*r* = −0.329, *P* < 0.05) and FAS (*r* = 0.345, *P* < 0.05), and between aggression and the RCS (*r* = −0.285, *P* < 0.05) and FAS (*r* = 0.391, *P* < 0.001). The total NPI‐Q score was significantly correlated with the RCS (*r* = −0.301, *P* < 0.05) and FAS (*r* = 0.378, *P* < 0.001). However, depression and irritability only showed significant correlations with the FAS (*r* = 0.264, *P* < 0.05; *r* = 0.469, *P* < 0.001).

**Table 2:** Correlations between the Family Attitude Scale (FAS) and Relationship Closeness Scale (RCS) at T1 and the Neuropsychiatric Inventory Questionnaire (NPI‐Q) at T2 (*N* = 56)

NPI‐Q item	RCS^a^	FAS^b^
Delusion	−0.329^c^	0.345^d^
Hallucination	0.003	0.048
Aggression	−0.285^c^	0.391^d^
Depression	−0.247	0.264^c^
Anxiety	0.020	−0.034
Elation	0.027	−0.200
Apathy	−0.195	0.171
Disinhibition	−0.019	0.101
Irritability	−0.2488	0.469^d^
Aberrant motor activity	0.003	0.000
Night‐time behaviour	−0.105	0.142
Appetite/eating change	−0.206	0.218
Total	−0.301^c^	0.378^d^
RCS	1	−0.671^d^

^a^ The RCS at T1.

^b^ The FAS at T2.

^c^ *P* < 0.05.

^d^ *P* < 0.001.

### Mediation

To test the mediation hypothesis, PROCESS macro (Model 4) was used to examine the mediating effect of EE. Table 3 shows that the indirect effect of RCS on BPSD was significant (95% CI = −0.6097, −0.1790, where the 95% CI excluded 0). Meanwhile, direct effects were not significant (95% CI = −0.6124, 0.3673), indicating that the FAS mediated the relationship between the RCS and BPSD, with a mediating effect of 71%. The FAS also mainly mediated the relationship between the RCS and aggression (95% CI = −0.1514, −0.00048, excluding 0), and the direct effect was not significant. Because the correlation analysis did not show a significant relationship between the RCS, FAS, and other domains, we did not perform a mediation analysis for these variables.

**Table 3:** Mediating effect of the Family Attitude Scale on the relationship between the total behavioural and psychological symptoms of dementia (BPSD) and Relationship Closeness Scale (RCS) (*N* = 56)

	Effect	SE	95% CI	
			Lower	Upper
RCS and BPSD^b^				
Total	−0.4298	0.1853	−0.8013	−0.0584
Direct	−0.1225	0.2442	−0.6124	0.3673
Indirect (mediation)	−0.3073	0.1490	−0.6097	−0.1790
RCS^a^ and delusion				
Total	−0.0736	0.0288	0.0134	−0.1314
Direct	−0.0396	0.0386	−0.1170	0.0377
Indirect (mediation)	−0.0340	0.0297	−0.0933	0.0238
RCS^a^ and aggression				
Total	−0.0856	0.0392	−0.1641	−0.0070
Direct	−0.0123	0.0512	−0.1150	0.0903
Indirect (mediation)	−0.0732	0.0370	−0.1514	−0.0048

^a^ The Relationship closeness scale.

^b^ Behavioral and Psychological Symptoms of Dementia.

## DISCUSSION

This study conducted a mediation analysis to explore how sociodemographic and behavioural characteristics of caregivers relate to their relationship closeness with people with dementia, caregiver EE, and BPSD. The results indicated a significant correlation between caregiver EE, intimacy level, and BPSD in people with dementia. Caregiver EE significantly moderated their intimacy level with people with dementia and BPSD. Further exploration revealed that higher intimacy levels were associated with reduced aggression in people with dementia.

Depression was the most common type of BPSD at both time points, while disinhibition was the least common. Prior research in Japan indicated that agitation/aggression was the most common, whereas elation/euphoria was the least.^26^ Similarly, Ikeda *et al*.^27^ reported that a majority of community‐dwelling individuals with dementia exhibited at least one type of BPSD, with apathy and agitation/aggression being particularly prevalent. Additional studies noted that circadian rhythm disturbances, delusions, and care resistance were more common among elderly individuals with dementia receiving home‐visit medical care.^28^ Discrepancies in findings across studies may stem from differences in sample size, dementia severity, and assessment tools.

Cultural values, particularly in East Asian societies, play a critical role in shaping caregiving experiences and outcomes. The concept of filial piety emphasises family responsibilities, moral obligations, and the preservation of family harmony.^29^ While this cultural value can promote close family connections, it can also increase caregiver stress. Caregivers may suppress their emotional distress to maintain family harmony, which may lead to higher levels of EE and increase the difficulty of managing the challenging behaviours of patients.

Japanese cultural norms generally emphasise family harmony and discourage open expression of dissatisfaction. Caregivers may hinder the use of external support services (such as nursing homes) to meet the value of filial piety.^30^ However, when family members have to use external services to reduce the burden, it may also cause disagreements among family members, which often leads to damage to family relationships.^31^

Cultural values also play a pivotal role in shaping caregiver–patient relationships, further influencing caregiving outcomes. Although this study did not find a direct relationship between closeness and BPSD, insights from other cultural contexts remain valuable. Previous studies in England have shown that higher EE in caregivers is associated with the exacerbation of symptoms in people with dementia.^11^ According to Eifert *et al*.,^32^ caregiver–patient relationships often stem from pre‐existing family roles, obligations, and a strong sense of responsibility. In long‐term care, these relationships evolve through identity reconstruction, replacing earlier interaction patterns. Social expectations, including family duties and gender norms, further shape caregiving as a moral obligation rather than a personal choice. We speculate that in Japanese society, close relationships between caregivers and people may be more influenced by a sense of responsibility and family obligations rather than emotional intimacy.

Caregivers' negative outcomes, including care burden and depression, were strongly associated with BPSD, such as restlessness, aggression, and irritability. These outcomes appear to be mediated through EE, particularly by cultural expectations such as filial piety, which emphasises attention and responsibility for older family members.^9^ While these findings suggest that cultural expectations may moderate the relationship between caregiver–patient closeness and caregiving outcomes, evidence from other contexts highlights the universal importance of close caregiver–patient relationships. A longitudinal study in the United Kingdom indicated that, among community‐dwelling elderly people with dementia and their caregivers, individuals who reported closer relationships experienced a smaller increase in the severity of neuropsychiatric symptoms over time.^33^ These findings suggest that caregiving outcomes benefit from relational closeness, although the mechanisms may vary depending on cultural contexts.

EE conveys the attitudes of caregivers toward people with dementia in the caregiving process, as demonstrated in this study, where EE was associated with the total NPI‐Q score, delusions, irritability, depression, and abnormal motor behaviour scores at T2. The results indicate that higher levels of EE among caregivers are linked to the exacerbation of BPSD, possibly because of their perception that inappropriate behaviours exhibited by the people with dementia are intentional rather than disease induced.^34^ Therefore, caregivers tend to employ critical measures to ensure patient safety or to correct inappropriate behaviours.^35^

This study was conducted over a short duration and had a limited sample size. Moreover, data were collected in specific cities within Osaka, which may reflect the perspectives of a particular demographic group rather than a broader population. This introduces potential biases, such as selection bias, that limit the generalisability of the findings.^36^ While there is limited research on the impact of Osaka's unique culture on caregiver–patient relationships, residents of the city are generally described as warm, outgoing, and community‐oriented. These cultural characteristics may promote open communication and a collective approach to care, which may mitigate EE and mitigate its impact on BPSD. Nevertheless, it is worth noting that this study utilised longitudinal mediation analysis, a methodology well‐suited to smaller sample sizes. The analysis achieved an intraclass correlation coefficient (ICC) of 0.7. Based on prior research on sample size thresholds for ICC values of 0.7, the sample size in this study falls within an appropriate range.^37^

Despite these limitations, the study's strengths include its use of a longitudinal mediation analysis, which highlight the importance of addressing emotional and relational dynamics in non‐pharmacological interventions for dementia care. Future research should explore how cultural values shape caregiving outcomes, caregiver EE, and caregiver–patient relationships to inform tailored interventions that address both cultural and individual needs.

## Data Availability

The data that support the findings of this study are available on request from the corresponding author. The data are not publicly available due to privacy or ethical restrictions.
